# 1-[(*E*)-4-(5-Bromo-1*H*-indol-3-yl)-1-methyl-2,5,6,7-tetra­hydro-1*H*-azepin-2-yl­idene]propan-2-one

**DOI:** 10.1107/S1600536810019975

**Published:** 2010-06-05

**Authors:** Madeleine Helliwell, Masomeh Aghazadeh, Mehdi M. Baradarani, John A. Joule

**Affiliations:** a The School of Chemistry, The University of Manchester, Manchester M13 9PL, England; b Department of Chemistry, Faculty of Science, University of Urmia, Urmia 57135, Iran

## Abstract

In the title compound, C_18_H_19_BrN_2_O, the seven-membered azepine ring adopts a twist-boat conformation: the bond angles about the azepine N atom are indicative of *sp* ^2^ hybridization. The dihedral angle between the plane of the carbon–carbon double bond of the enone unit and the mean plane of the indole ring is 27.8 (1)°. In the crystal, an N—H⋯O hydrogen bond links the mol­ecules into chains along the *b* axis.

## Related literature

For structure intrepretation tools, see: Allen (2002 ▶); Allen *et al.* (1993 ▶); Cremer & Pople (1975 ▶). For the reaction chemistry of (*Z*)-3-(1-methyl­pyrrolidin-2-yl­idene)-3*H*-indole, see: Bishop *et al.* (1981*a* ▶,*b* ▶, 1982*a* ▶,**b* ▶)*; Harris & Joule (1978*a* ▶,*b* ▶). 
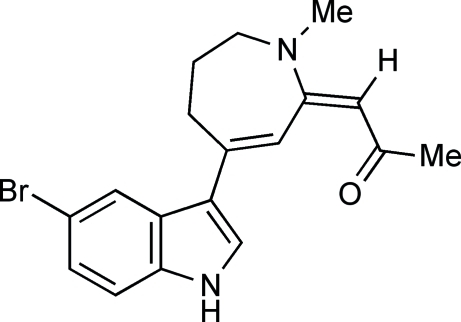


## Experimental

### 

#### Crystal data

C_18_H_19_BrN_2_O
*M* *_r_* = 359.26
Monoclinic, 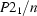

*a* = 14.496 (2) Å
*b* = 6.6677 (10) Å
*c* = 16.372 (3) Å
β = 90.267 (2)°
*V* = 1582.4 (4) Å^3^
*Z* = 4
Mo *K*α radiation
μ = 2.60 mm^−1^
*T* = 100 K
0.30 × 0.20 × 0.20 mm

#### Data collection

Bruker APEX CCD area-detector diffractometer
Absorption correction: multi-scan (*SADABS*; Sheldrick, 1996 ▶) *T*_min_ = 0.829, *T*_max_ = 1.000
12098 measured reflections
3239 independent reflections
3021 reflections with *I* > 2σ(*I*)
*R*_int_ = 0.023

#### Refinement

*R*[*F* ^2^ > 2σ(*F* ^2^)] = 0.027
*wR*(*F* ^2^) = 0.069
*S* = 1.25
3239 reflections
205 parameters
H atoms treated by a mixture of independent and constrained refinement
Δρ_max_ = 0.39 e Å^−3^
Δρ_min_ = −0.23 e Å^−3^

### 

Data collection: *SMART* (Bruker, 2001 ▶); cell refinement: *SAINT* (Bruker, 2002 ▶); data reduction: *SAINT*; program(s) used to solve structure: *SHELXS97* (Sheldrick, 2008 ▶); program(s) used to refine structure: *SHELXL97* (Sheldrick, 2008 ▶); molecular graphics: *SHELXTL* (Sheldrick, 2008 ▶) and *PLATON* (Spek, 2009 ▶); software used to prepare material for publication: *SHELXTL* and *PLATON*.

## Supplementary Material

Crystal structure: contains datablocks global, I. DOI: 10.1107/S1600536810019975/jj2030sup1.cif

Structure factors: contains datablocks I. DOI: 10.1107/S1600536810019975/jj2030Isup2.hkl

Additional supplementary materials:  crystallographic information; 3D view; checkCIF report

## Figures and Tables

**Table 1:** Hydrogen-bond geometry (Å, °)

*D*—H⋯*A*	*D*—H	H⋯*A*	*D*⋯*A*	*D*—H⋯*A*
N1—H1*N*⋯O1^i^	0.78 (2)	2.02 (2)	2.7549 (19)	159 (2)

Symmetry code: (i) 
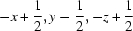
.

**Fig. 1.**
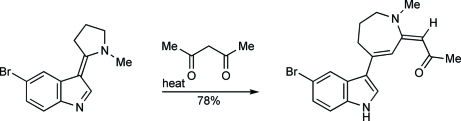
The reaction scheme for 5-bromo-3-(1-methylpyrrolidin-2-ylidene)-3H -indole with pentane-2,4-dione to form (I), C18H19BrN2.

**Fig. 2.**
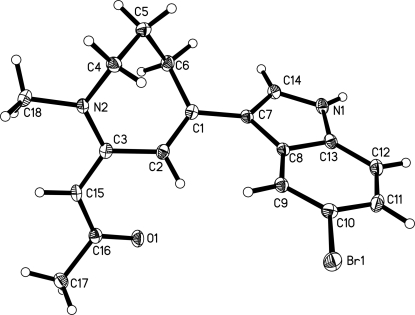
The structure of (I) with displacement ellipsoids for the non-hydrogen atoms drawn at the 50% probability level.

**Fig. 3.**
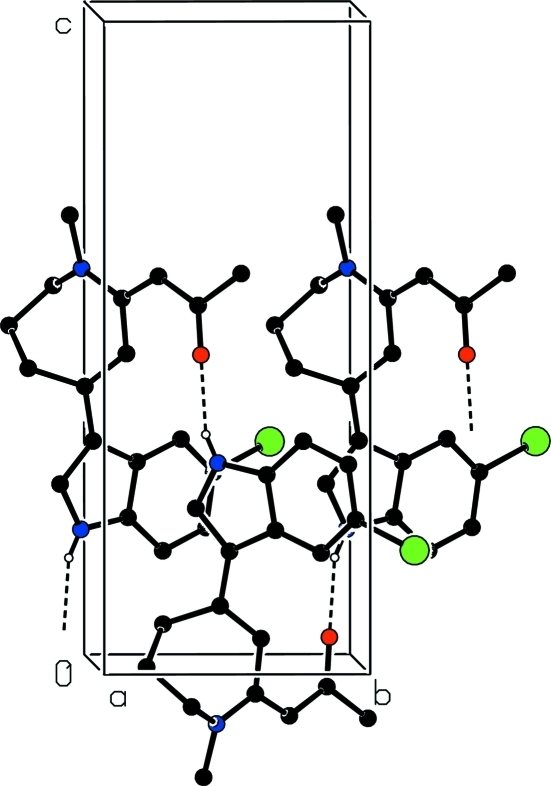
Packing arrangement of (I) viewed down a. Dashed lines indicate N—H···O hydrogen bonds between the indole ring and the carbonyl group extending from the propan-2-one group linking the molecules into chains along the c axis.

## supplementary crystallographic information

### Comment

Study of the reaction chemistry of (Z)-3-(1-methylpyrrolidin-2-ylidene)-3H-indole has revealed some remarkable properties and transformations. For example, it was shown to be a remarkably strong base, pK_a_ 10.6, for an imine, to be compared with that for 4a-methyl-1,2,3,4-tetrahydro-4a(i)H-carbazole with a pK_a_ of 3.6 (Harris and Joule, 1978a,b). It reacts with pentane-2,4-dione to give ((E)-4-(1H-indol-3-yl)- 2,5,6,7-tetrahydro-1-methyl-1H-azepin-2-ylidene)propan-2-one via an extensive rearrangement (Bishop et al., 1981a,b). In addition, it reacts with diethyl malonate giving 7-(2-aminophenyl)-5-ethoxycarbonyl-2,3-dihydro-4-hydroxy-1-methylindole, involving another extensive and unprecedented rearrangement (Bishop et al., 1982a,b). We detail here the crystal structure of the product ,(I), C_18_H_19_BrN_2_, formed by reacting (Z)-5-bromo-3-(1-methylpyrrolidin-2-ylidene)-3H-indole with pentane-2,4-dione following the procedure for the des-bromo-prototype (Bishop et al., 1981a,b), (Fig. 1), leading to a 5-bromoindol-3-yl-substituted tetrahydroazepine, (I).

The seven-membered azepine ring adopts a twist-boat conformation as shown by the puckering parameters (Cremer & Pople, 1975; Allen et al., 1993) q_2_= 1.008 (2); q_3_= 0.176 (2); φ_2_= 298.0 (1); φ_3_ = 34.4 (6) ° (Spek, 2009), Fig.2.  Bond distances and angles in (I) are in the normal range (Allen, 2002).  The planar 5-bromoindole bicycle is not coplanar with the enone in the seven-membered azepine ring.  The dihedral angle between the enone double bond and the mean plane of the indole ring is 27.8 (1) °.  The azepine nitrogen is sp^2^ hybridized, with the sum of the angles around it being 359.4 °, indicating its conjugating interaction with the exocyclic enone, i.e. it is a vinylogous amide nitrogen. The exocyclic double bond has E geometry.  A N1—H1···O1 hydrogen bond between the indole ring and the carbonyl group extending from the propan-2-one group links the molecules into chains along the b axis (Fig. 3).

### Experimental

(*Z*)-5-Bromo-3-(1-methylpyrrolidin-2-ylidene)-3*H*-indole (0.5 g, 1.8 mmol) was heated in refluxing pentane-2,4-dione (11 ml) for 4 h (Fig. 1). When excess diketone was removed by distillation under vacuum, a yellow solid was obtained which was partitioned between 2*M* HCl and ethyl acetate. The basic product was isolated from the aqueous acidic layer by basification with potassium carbonate and extraction with dichloromethane (0.48 g, 78%). The product was recrystallized in *n*-hexane/ethanol to give yellow crystalline material, mp 459–461 K.

### Refinement

H atoms bonded to C were included in calculated positions using the riding method, with aromatic, methylene and methyl C—H distances of 0.98, 0.99 and 0.95 Å, respectively and U~eq ~values 1.2 and 1.5 times those of the parent atoms; the torsion angles of the methyl H atoms were optimized to give the best fit to the electron density. The H atom bonded to N1 was found by difference Fourier methods and refined isotropically with N–H = 0.78 (2) Å.

### Crystal data

**Table:**

C_18_H_19_BrN_2_O	*F*(000) = 736
*M**_r_* = 359.26	*D*_x_ = 1.508 Mg m^−^^3^
Monoclinic, *P*2_1_/*n*	Melting point = 459–461 K
Hall symbol: -P 2yn	Mo *K*α radiation, λ = 0.71073 Å
*a* = 14.496 (2) Å	Cell parameters from 915 reflections
*b* = 6.6677 (10) Å	θ = 2.8–26.4°
*c* = 16.372 (3) Å	µ = 2.60 mm^−^^1^
β = 90.267 (2)°	*T* = 100 K
*V* = 1582.4 (4) Å^3^	Block, yellow
*Z* = 4	0.30 × 0.20 × 0.20 mm

### Data collection

**Table:**

Bruker APEX CCD area-detector diffractometer	3239 independent reflections
Radiation source: fine-focus sealed tube	3021 reflections with *I* > 2σ(*I*)
graphite	*R*_int_ = 0.023
Detector resolution: 8.33 pixels mm^-1^	θ_max_ = 26.4°, θ_min_ = 1.9°
φ and ω scans	*h* = −17→18
Absorption correction: multi-scan (*SADABS*; Sheldrick, 1996)	*k* = −8→8
*T*_min_ = 0.829, *T*_max_ = 1.000	*l* = −20→20
12098 measured reflections	

### Refinement

**Table:**

Refinement on *F*^2^	Primary atom site location: structure-invariant direct methods
Least-squares matrix: full	Secondary atom site location: difference Fourier map
*R*[*F*^2^ > 2σ(*F*^2^)] = 0.027	Hydrogen site location: inferred from neighbouring sites
*wR*(*F*^2^) = 0.069	H atoms treated by a mixture of independent and constrained refinement
*S* = 1.25	*w* = 1/[σ^2^(*F*_o_^2^) + (0.0342*P*)^2^ + 0.3299*P*] where *P* = (*F*_o_^2^ + 2*F*_c_^2^)/3
3239 reflections	(Δ/σ)_max_ = 0.031
205 parameters	Δρ_max_ = 0.39 e Å^−^^3^
0 restraints	Δρ_min_ = −0.23 e Å^−^^3^

### Special details

**Table:**

Experimental. ^1^H-NMR (CDCl_3_) δ (ppm) 2.0 (2H, qn, *J* = 6.6 Hz, azepin-6-yl-H_2_), 2.18 (3H, s, MeCO overlying 2H, m, azepin-5-yl-H_2_),), 3.14 (1H, s, MeN), 3.41 (2H, t, *J* = 6.3 Hz, azepin-7-yl-H_2_), 5.19 (1H, s, exocyclic =CH), 6.35 (1H, s, azepin-3-yl-H), 7.0 (1H, d, *J* = 8.7 Hz, ArH), 7.13 (1H, d, *J* = 8.7 Hz, ArH), 7.23 (1H, s, indol-4-yl-H), 7.93 (1H, s, indol-2-yl-H), 11.12 (1H, bs, NH). ^13^C-NMR (CDCl_3_) δ 28.8, 31.1, 31.4, 39.7, 52.2, 94.6, 113.4, 113.6, 119.6, 122.4, 124.2, 126.4, 127.1, 134.5, 141.3, 163.9, 193.4. ν_max_ 2915, 1611, 1506, 1340, 1189, 972, 787. λ_max_ (EtOH) 236, 261, 350 nm.
Geometry. All e.s.d.'s (except the e.s.d. in the dihedral angle between two l.s. planes) are estimated using the full covariance matrix. The cell e.s.d.'s are taken into account individually in the estimation of e.s.d.'s in distances, angles and torsion angles; correlations between e.s.d.'s in cell parameters are only used when they are defined by crystal symmetry. An approximate (isotropic) treatment of cell e.s.d.'s is used for estimating e.s.d.'s involving l.s. planes.
Refinement. Refinement on *F*^2^ against ALL reflections. Weighted *R*-factors *wR* and all goodnesses of fit *S* are based on *F*^2^, conventional *R*-factors *R* are based on *F*, with *F* set to zero for negative *F*^2^. The threshold expression of *F*^2^ > σ(*F*^2^) is used only for calculating *R*-factors(gt) *etc*. and is not relevant to the choice of reflections for refinement. *R*-factors based on *F*^2^ are statistically about twice as large as those based on *F*, and *R*- factors based on ALL data will be even larger.

### Fractional atomic coordinates and isotropic or equivalent isotropic displacement parameters (Å^2^)

**Table:**

	*x*	*y*	*z*	*U*_iso_*/*U*_eq_	
Br1	0.545967 (12)	1.20143 (3)	0.175612 (11)	0.01975 (8)	
O1	0.13649 (9)	0.9119 (2)	0.03015 (7)	0.0188 (3)	
N1	0.35260 (10)	0.4764 (2)	0.30389 (9)	0.0153 (3)	
N2	0.29940 (10)	0.4751 (2)	−0.09752 (9)	0.0140 (3)	
C1	0.30698 (11)	0.4879 (3)	0.08357 (10)	0.0130 (3)	
C2	0.28922 (11)	0.6432 (3)	0.03283 (10)	0.0135 (3)	
H2	0.2962	0.7749	0.0542	0.016*	
C3	0.26004 (11)	0.6240 (3)	−0.05267 (10)	0.0131 (3)	
C4	0.38380 (12)	0.3749 (3)	−0.06878 (10)	0.0161 (4)	
H4A	0.4246	0.4753	−0.0425	0.019*	
H4B	0.4169	0.3188	−0.1163	0.019*	
C5	0.36434 (14)	0.2070 (3)	−0.00819 (11)	0.0179 (4)	
H5A	0.4223	0.1688	0.0200	0.021*	
H5B	0.3410	0.0879	−0.0379	0.021*	
C6	0.29263 (13)	0.2741 (3)	0.05526 (11)	0.0165 (4)	
H6A	0.2301	0.2616	0.0311	0.020*	
H6B	0.2960	0.1836	0.1031	0.020*	
C7	0.33667 (11)	0.5212 (3)	0.16813 (10)	0.0122 (3)	
C8	0.38650 (11)	0.6899 (2)	0.20169 (10)	0.0118 (3)	
C9	0.43010 (11)	0.8588 (3)	0.16825 (10)	0.0135 (3)	
H9	0.4274	0.8853	0.1113	0.016*	
C10	0.47689 (12)	0.9849 (3)	0.22064 (11)	0.0151 (3)	
C11	0.47997 (12)	0.9571 (3)	0.30543 (11)	0.0180 (4)	
H11	0.5106	1.0521	0.3393	0.022*	
C12	0.43834 (13)	0.7910 (3)	0.33961 (11)	0.0170 (4)	
H12	0.4396	0.7689	0.3969	0.020*	
C13	0.39445 (11)	0.6568 (3)	0.28686 (10)	0.0138 (3)	
C14	0.31950 (11)	0.3958 (3)	0.23342 (10)	0.0146 (3)	
H14	0.2887	0.2703	0.2296	0.017*	
C15	0.19750 (12)	0.7566 (3)	−0.08831 (10)	0.0147 (3)	
H15	0.1897	0.7498	−0.1459	0.018*	
C16	0.14431 (11)	0.9017 (3)	−0.04539 (10)	0.0143 (3)	
C17	0.09459 (12)	1.0600 (3)	−0.09551 (11)	0.0181 (4)	
H17A	0.0310	1.0732	−0.0762	0.027*	
H17B	0.0941	1.0201	−0.1531	0.027*	
H17C	0.1265	1.1887	−0.0896	0.027*	
C18	0.26809 (13)	0.4286 (3)	−0.18007 (10)	0.0183 (4)	
H18A	0.2007	0.4375	−0.1826	0.027*	
H18B	0.2875	0.2925	−0.1946	0.027*	
H18C	0.2950	0.5246	−0.2185	0.027*	
H1N	0.3516 (15)	0.431 (3)	0.3474 (14)	0.023 (6)*	

### Atomic displacement parameters (Å^2^)

**Table:**

	*U*^11^	*U*^22^	*U*^33^	*U*^12^	*U*^13^	*U*^23^
Br1	0.02014 (12)	0.01765 (12)	0.02146 (12)	−0.00671 (7)	−0.00231 (8)	0.00178 (7)
O1	0.0215 (6)	0.0241 (7)	0.0106 (6)	0.0039 (5)	−0.0001 (5)	−0.0015 (5)
N1	0.0172 (7)	0.0186 (8)	0.0102 (7)	−0.0021 (6)	−0.0003 (6)	0.0039 (6)
N2	0.0162 (7)	0.0159 (7)	0.0099 (7)	0.0025 (6)	−0.0014 (5)	−0.0004 (6)
C1	0.0106 (8)	0.0158 (8)	0.0125 (8)	−0.0015 (6)	0.0008 (6)	−0.0009 (7)
C2	0.0116 (8)	0.0157 (8)	0.0132 (8)	−0.0010 (6)	−0.0004 (6)	−0.0017 (7)
C3	0.0131 (8)	0.0146 (8)	0.0116 (8)	−0.0038 (7)	0.0006 (6)	0.0005 (7)
C4	0.0178 (9)	0.0164 (9)	0.0140 (8)	0.0028 (7)	0.0012 (7)	0.0013 (7)
C5	0.0249 (10)	0.0130 (9)	0.0158 (9)	0.0016 (7)	−0.0007 (8)	−0.0005 (7)
C6	0.0218 (9)	0.0151 (9)	0.0126 (8)	−0.0036 (7)	−0.0010 (7)	0.0013 (7)
C7	0.0097 (8)	0.0147 (8)	0.0122 (8)	0.0019 (6)	0.0000 (6)	0.0001 (6)
C8	0.0099 (8)	0.0143 (8)	0.0112 (8)	0.0029 (6)	0.0002 (6)	−0.0004 (6)
C9	0.0124 (8)	0.0160 (8)	0.0121 (8)	0.0016 (7)	0.0000 (6)	0.0000 (7)
C10	0.0127 (8)	0.0140 (8)	0.0186 (9)	−0.0002 (6)	−0.0002 (7)	0.0012 (7)
C11	0.0180 (9)	0.0200 (9)	0.0160 (9)	−0.0019 (7)	−0.0037 (7)	−0.0040 (7)
C12	0.0170 (9)	0.0217 (10)	0.0124 (8)	0.0018 (7)	−0.0017 (7)	−0.0007 (7)
C13	0.0109 (8)	0.0174 (8)	0.0129 (8)	0.0011 (7)	−0.0002 (6)	0.0011 (7)
C14	0.0133 (8)	0.0160 (9)	0.0144 (8)	−0.0002 (7)	−0.0009 (6)	0.0006 (7)
C15	0.0159 (9)	0.0191 (8)	0.0092 (8)	−0.0011 (7)	−0.0023 (7)	0.0006 (7)
C16	0.0123 (8)	0.0161 (9)	0.0145 (8)	−0.0028 (7)	−0.0015 (6)	−0.0001 (7)
C17	0.0184 (9)	0.0200 (9)	0.0158 (9)	0.0028 (7)	−0.0012 (7)	0.0018 (7)
C18	0.0230 (9)	0.0185 (9)	0.0134 (8)	−0.0002 (7)	−0.0013 (7)	−0.0038 (7)

### Geometric parameters (Å, °)

**Table:**

Br1—C10	1.9073 (17)	C7—C14	1.381 (2)
O1—C16	1.244 (2)	C7—C8	1.444 (2)
N1—C14	1.358 (2)	C8—C9	1.404 (2)
N1—C13	1.376 (2)	C8—C13	1.416 (2)
N1—H1N	0.78 (2)	C9—C10	1.377 (2)
N2—C3	1.362 (2)	C9—H9	0.9500
N2—C18	1.457 (2)	C10—C11	1.401 (2)
N2—C4	1.469 (2)	C11—C12	1.381 (3)
C1—C2	1.352 (2)	C11—H11	0.9500
C1—C7	1.465 (2)	C12—C13	1.395 (2)
C1—C6	1.513 (2)	C12—H12	0.9500
C2—C3	1.466 (2)	C14—H14	0.9500
C2—H2	0.9500	C15—C16	1.424 (2)
C3—C15	1.393 (2)	C15—H15	0.9500
C4—C5	1.523 (2)	C16—C17	1.517 (2)
C4—H4A	0.9900	C17—H17A	0.9800
C4—H4B	0.9900	C17—H17B	0.9800
C5—C6	1.540 (3)	C17—H17C	0.9800
C5—H5A	0.9900	C18—H18A	0.9800
C5—H5B	0.9900	C18—H18B	0.9800
C6—H6A	0.9900	C18—H18C	0.9800
C6—H6B	0.9900		
			
C14—N1—C13	109.16 (15)	C13—C8—C7	106.97 (15)
C14—N1—H1N	128.0 (17)	C10—C9—C8	117.91 (15)
C13—N1—H1N	122.8 (17)	C10—C9—H9	121.0
C3—N2—C18	121.71 (14)	C8—C9—H9	121.0
C3—N2—C4	120.60 (14)	C9—C10—C11	123.34 (16)
C18—N2—C4	117.12 (14)	C9—C10—Br1	118.64 (13)
C2—C1—C7	121.26 (15)	C11—C10—Br1	117.95 (13)
C2—C1—C6	120.54 (15)	C12—C11—C10	119.71 (16)
C7—C1—C6	118.15 (15)	C12—C11—H11	120.1
C1—C2—C3	124.97 (16)	C10—C11—H11	120.1
C1—C2—H2	117.5	C11—C12—C13	117.54 (17)
C3—C2—H2	117.5	C11—C12—H12	121.2
N2—C3—C15	120.71 (15)	C13—C12—H12	121.2
N2—C3—C2	117.31 (15)	N1—C13—C12	129.44 (16)
C15—C3—C2	121.94 (15)	N1—C13—C8	107.56 (15)
N2—C4—C5	112.73 (15)	C12—C13—C8	123.00 (16)
N2—C4—H4A	109.0	N1—C14—C7	110.71 (16)
C5—C4—H4A	109.0	N1—C14—H14	124.6
N2—C4—H4B	109.0	C7—C14—H14	124.6
C5—C4—H4B	109.0	C3—C15—C16	125.26 (16)
H4A—C4—H4B	107.8	C3—C15—H15	117.4
C4—C5—C6	110.69 (14)	C16—C15—H15	117.4
C4—C5—H5A	109.5	O1—C16—C15	125.42 (16)
C6—C5—H5A	109.5	O1—C16—C17	117.02 (15)
C4—C5—H5B	109.5	C15—C16—C17	117.55 (15)
C6—C5—H5B	109.5	C16—C17—H17A	109.5
H5A—C5—H5B	108.1	C16—C17—H17B	109.5
C1—C6—C5	112.85 (15)	H17A—C17—H17B	109.5
C1—C6—H6A	109.0	C16—C17—H17C	109.5
C5—C6—H6A	109.0	H17A—C17—H17C	109.5
C1—C6—H6B	109.0	H17B—C17—H17C	109.5
C5—C6—H6B	109.0	N2—C18—H18A	109.5
H6A—C6—H6B	107.8	N2—C18—H18B	109.5
C14—C7—C8	105.58 (15)	H18A—C18—H18B	109.5
C14—C7—C1	125.92 (16)	N2—C18—H18C	109.5
C8—C7—C1	128.47 (15)	H18A—C18—H18C	109.5
C9—C8—C13	118.31 (15)	H18B—C18—H18C	109.5
C9—C8—C7	134.56 (16)		
			
C7—C1—C2—C3	179.45 (15)	C13—C8—C9—C10	−1.1 (2)
C6—C1—C2—C3	−3.2 (3)	C7—C8—C9—C10	−175.93 (18)
C18—N2—C3—C15	−9.5 (2)	C8—C9—C10—C11	−2.6 (3)
C4—N2—C3—C15	161.62 (16)	C8—C9—C10—Br1	174.36 (12)
C18—N2—C3—C2	173.07 (15)	C9—C10—C11—C12	3.2 (3)
C4—N2—C3—C2	−15.8 (2)	Br1—C10—C11—C12	−173.73 (14)
C1—C2—C3—N2	−38.6 (2)	C10—C11—C12—C13	0.0 (3)
C1—C2—C3—C15	144.00 (18)	C14—N1—C13—C12	−179.52 (18)
C3—N2—C4—C5	83.4 (2)	C14—N1—C13—C8	0.26 (19)
C18—N2—C4—C5	−105.10 (17)	C11—C12—C13—N1	176.04 (18)
N2—C4—C5—C6	−44.4 (2)	C11—C12—C13—C8	−3.7 (3)
C2—C1—C6—C5	70.7 (2)	C9—C8—C13—N1	−175.47 (15)
C7—C1—C6—C5	−111.84 (17)	C7—C8—C13—N1	0.68 (19)
C4—C5—C6—C1	−40.5 (2)	C9—C8—C13—C12	4.3 (3)
C2—C1—C7—C14	150.24 (18)	C7—C8—C13—C12	−179.53 (16)
C6—C1—C7—C14	−27.2 (2)	C13—N1—C14—C7	−1.2 (2)
C2—C1—C7—C8	−27.8 (3)	C8—C7—C14—N1	1.53 (19)
C6—C1—C7—C8	154.82 (17)	C1—C7—C14—N1	−176.84 (15)
C14—C7—C8—C9	173.91 (19)	N2—C3—C15—C16	172.13 (16)
C1—C7—C8—C9	−7.8 (3)	C2—C3—C15—C16	−10.5 (3)
C14—C7—C8—C13	−1.33 (18)	C3—C15—C16—O1	−10.6 (3)
C1—C7—C8—C13	176.99 (16)	C3—C15—C16—C17	168.01 (16)

### Hydrogen-bond geometry (Å, °)

**Table:**

*D*—H···*A*	*D*—H	H···*A*	*D*···*A*	*D*—H···*A*
N1—H1N···O1^i^	0.78 (2)	2.02 (2)	2.7549 (19)	159 (2)

Symmetry codes: (i) −*x*+1/2, *y*−1/2, −*z*+1/2.
